# Plants’ Epigenetic Mechanisms and Abiotic Stress

**DOI:** 10.3390/genes12081106

**Published:** 2021-07-21

**Authors:** Matin Miryeganeh

**Affiliations:** Plant Epigenetics Unit, Okinawa Institute of Science and Technology Graduate University, 1919-1 Tancha, Onna-son, Okinawa 904-0412, Japan; matin.miryeganeh@oist.jp; Tel.: +81-80-4207-4224

**Keywords:** DNA methylation, histone modification, plant epigenetics, abiotic stress, stress memory, salinity stress, heat stress, drought stress

## Abstract

Plants are sessile organisms that need to adapt to constantly changing environmental conditions. Unpredictable climate change places plants under a variety of abiotic stresses. Studying the regulation of stress-responsive genes can help to understand plants’ ability to adapt to fluctuating environmental conditions. Changes in epigenetic marks such as histone modifications and DNA methylation are known to regulate gene expression by their dynamic variation in response to stimuli. This can then affect their phenotypic plasticity, which helps with the adaptation of plants to adverse conditions. Epigenetic marks may also provide a mechanistic basis for stress memory, which enables plants to respond more effectively and efficiently to recurring stress and prepare offspring for potential future stresses. Studying epigenetic changes in addition to genetic factors is important to better understand the molecular mechanisms underlying plant stress responses. This review summarizes the epigenetic mechanisms behind plant responses to some main abiotic stresses.

## 1. Overview

Unpredictable climate changes cause plants to often be exposed to various abiotic stresses that mainly include extreme temperature, dehydration, high salinity, low nutrition, ultraviolet radiation, and heavy metal toxicity, which all affect the productivity of plants. The dynamics of epigenetic codes play an important role in regulating genes in response to environmental stresses [1]. For example, histone modification and DNA methylation are known to alter the expression of stress-responsive genes at the transcriptional and post-transcriptional levels by changing the chromatin status of those genes [2]. For instance, transcription factors and RNA-directed DNA methylation (RdDM) play a key role in the regulation of gene expression under abiotic stress in plants. Some known heat stress genes are regulated through RdDM pathway-mediated DNA methylation [3]. As another example, the MYB74 transcription factor in the Arabidopsis plant is silenced by RdDM in normal conditions, and when the plant experiences salinity stress, it will become desilenced [4]. In addition, some epigenetic changes such as histone modifications play a key role in stress memory, which can be passed on to offspring [5].

How epigenetic marks affect gene expressions depends not only on the type of modification but also on their position on the genes, meaning, for example, if they are placed on the promoter region or gene body. For instance, when DNA methylation and H3K9me2 are positioned within the promoter region, they may prevent transcription and therefore repress the expression of genes, whereas there are cases where these heterochromatin epigenetic marks are located in the gene body and then help with the full-length transcription of the gene [6]. Transposable elements (TEs) often insert into the gene body of stress-related genes in plants’ genomes, and therefore, it is very likely that intragenic epigenetic modifications regulate the expression of stress-responsive genes, for example by affecting the alternative splicing of the transcripts [7]. Epigenetic changes usually return to their prestress state after the stimulus that was causing the stress is gone. However, it has been reported that in some cases, the epigenetic changes (or part of them) remain even after the withdrawal of stress and act as stress memory for plants. This stress memory then helps with their adaptation and even evolution in the longer term. Possible usage of this trait in plants can help to advance epigenome engineering in order to improve plants’ tolerance to environmental stresses and climate changes.

When genetically identical plants are exposed to various stresses, they show changes in DNA methylation. For example, apomictic *Taraxacum officinale* plants were exposed to some abiotic stresses, and all plants in all types of stresses showed significant variations in DNA methylation [8]. The results indicated that DNA methylation variation caused by stress is common and highlighted that epigenetic inheritance in the adaptation of plants can be independent of genetic variation among individuals. Many studies have also reported that stress-induced methylation patterns depend on the type of stress, the genotype, the tissue, and the organism, which also affect the regulation of a wide range of stress-responsive genes [9,10,11]. Another example is a comparative analysis of the methylome and gene expression in sixty annual clones of a stress-tolerant poplar genotype (*Populus simonii*), where the authors investigated the effect of four abiotic stress treatments (salinity, osmotic, heat, and cold) from 3 h to 24 h [12]. The DNA methylation level was higher after three hours of all stress treatments and was especially the highest for heat stress. The heat stress raised the methylation level to its maximum at six hours and maintained it unchanged for the rest of the treatment, whereas other stresses gradually raised the methylation levels up to 24 h. At the end of the treatment time (at 24 h), the DNA methylation levels were the highest for osmotic and cold treatment samples. Therefore, the patterns of methylated fragments were stress-specific, and different stress treatments showed different impacts on the expression of DNA methylation genes. In addition, some methylated fragments mapped to miRNAs and long noncoding RNAs. These noncoding RNAs and their putative target genes showed different expression patterns to stresses which could imply the effect of epigenetic regulation of noncoding RNA expression in the stress response of plants. This review summarizes some fundamental epigenetic regulations of stress-related genes in response to some main abiotic stresses such as heat, cold, salinity, and drought in plants.

## 2. Epigenetic and Heat Stress

The regulatory network of plant heat stress response has been recently reviewed [13]. Epigenetic mechanisms such as DNA methylation, histone modifications, histone variants, small RNAs, long noncoding RNAs, and other undefined epigenetic mechanisms can regulate the expression of genes in response to heat stress in order to protect plants against damage caused by extremely high temperature [14]. DNA methylation processes have been widely studied in response to heat stress, especially through the activity of methyltransferases. Studying the natural population of *Arabidopsis thaliana*, Shen et al. [15] identified several alleles linked to a plastic response to climate variation with diverse genome-wide CHH methylation associated with seasonality. They found a correlation between chromomethylase 2 (CMT2) and temperature seasonality and showed that cmt2 mutants are less sensitive to heat stress, suggesting that the genetic regulation of epigenetic modifications might be the underlying reason for natural adaptation to variable temperatures, potentially through differential allelic plasticity to heat stress [15]. In plants, DNA methylation can be directed by small RNAs (RNA-directed DNA methylation (RdDM)) using two plant-specific RNA polymerases—PolIV and PolV. Naydenov et al. [16] investigated the effect of heat stress on the expression of key DNA methylation genes, DNA methyltransferase (MET1, CMT3, and DRM2), the largest subunits of PoIIV (NRPD1), and PolV (NRPE1). They reported that the upregulation of these epigenetic modulators may be responsible for increased genome methylation in *Arabidopsis thaliana* under heat stress [16].

Heat shock proteins (HSPs) are also known to play important roles in heat tolerance in plants. Heat shock protein genes such as HSP18, HSP22.0, APX2, and HSP70 will accumulate histone H3 lysine 9 acetylation (H3K9Ac) and histone H3 lysine 4 trimethylation (H3K4me3) after a heat stress experience. In addition, histone modification and DNA methylation via the RdDM pathway help with plants’ heat tolerance as well [17,18]. While in the vegetative phase, *A. thaliana*’s imprinted gene SDC (suppressor of drm1, drm2, and cmt3), which is a target of the RdDM pathway, is silent by DNA methylation, it activates when plants experience heat stress in order to regulate the expression of genes, resulting in resistance to heat stress [19]. Thus, high-temperature stress might inactivate the silencing role of RdDM by the regulation of heat-tolerance genes [7]. Heat stress has been shown to epigenetically silence genes in *A. thaliana*. Popova et al. [3] tested the heat tolerance of a set of epigenetic mutants (mutants defective in DNA methylation, histone modifications, chromatin remodeling, or siRNA-based silencing pathways) and found that the transcriptional response to heat stress is dependent on the RNA-dependent DNA methylation pathway and also the Rpd3-type histone deacetylase HDA6. Their results also showed evidence that heat-dependent gene expression is influenced by nearby transposable elements [3].

Studying the molecular mechanism behind heat sensitivity and reduced seed size in rice, which is controlled by OsFIE1 (fertilization-independent endosperm), Folsom et al. [20] observed that DNA and histone methylation (H3K9me2) are the main factors regulating OsFIE, which is also temperature sensitive. They suggested that the thermal sensitivity of seed size in rice could be caused by changes in the epigenetic regulation of endosperm development [20]. Heer et al. [21] studied methylation patterns in Norway spruce (*Picea abies*) using four clone pairs, where clone members had grown in different climatic conditions for 24 years. They identified different gene body methylation patterns in different environments and reported differentially methylated positions (DMPs) between environments. This concluded that changes in methylation patterns are a possible pathway for a plant to respond to environmental changes. miRNAs also play an important role in epigenetic regulations by altering gene expression via the post-transcriptional silencing of complementary mRNA [14]. Changes in miRNA expression in response to heat stress in *Betula luminifera* [22] and in several poplar species [23,24] have previously been reported. Histone acetylation has also been reported under heat stress. A prototypical histone acetyltransferase (HAT) known as general control nonrepressed protein 5 (GCN5) is shown to be very important for heat-stress-responsive gene expression and thermotolerance in Arabidopsis. Hu et al. [25] showed that loss of function of GCN5 resulted in an acute deficiency of heat tolerance in Arabidopsis, and it negatively affected the regulation of heat thermotolerance genes. Mutations in the Arabidopsis histone acetyltransferase GCN5 gene resulted in reduced thermotolerance because of the deficit in the transcriptional activation of heat-stress-responsive genes such as HS transcription factors HSFA2 and HSFA3 and multiprotein bridging factor 1c (MBF1c) [14]. It is also known that the length of the heat stress period has different effects on epigenetic mechanisms, suggesting the complexity of the epigenetic regulation mechanism behind heat stress [14]. It has also been reported that during heat treatment, a histone variant H2A.Z alters the transcription of stress response genes [26].

## 3. Epigenetic and Cold Stress

Plants have a “cold acclimation” ability, meaning low-temperature (but nonfreezing) exposure improves their resistance to upcoming freezing conditions [27]. The C-REPEAT BINDING FACTOR (CBF)-COLD-RESPONSIVE (COR) pathway is among the well-characterized mechanisms in the plant cold stress response. Cold stress increases the levels of CBFs transcription factors, which then upregulate cold-responsive (COR) genes [28]. A chromatin remodeler gene named PICKLE (PKL) in Arabidopsis has been shown to participate in the CBF-dependent cold stress response and is necessary for efficient cold stress tolerance in plants [29]. *pkl* mutants are shown to be hypersensitive to cold stress. Analyses using *pkl* mutants under cold stress showed significant changes in the transcription level of CBF3, which is a key transcription factor for the regulation of cold-tolerance genes. The transcription factor gene CBF3 and downstream COR family genes such as COR15B and RD29A were downregulated in the *pkl* mutants after cold treatment, and H3K27me3 deposition was shown in COR genes [30]. PICKLE (PKL) is a subunit of the Mi-2/CHD3 subfamily of ATP-dependent chromatin remodelers important for cold acclimation. PICKLE plays a role in the regulation of the RdDM pathway [31] and also helps with the deposition of H3K27me3 [32], which suggests that both H3K27me3 and DNA methylation may affect cold-tolerance stress memory in plants. It has also been reported that cold stress reduces H3K27me3 in cold response genes, which was even maintained after the temperature returned to normal, supporting the idea that H3K27me3 may help with stress memory [30]. Thus, the PKL gene might influence plant response to cold stress by regulating the H3K27me3-dependent chromatin status of cold response genes [7].

HOS15 is a WD40 repeat-containing protein that is involved in histone deacetylation and cold tolerance and functions as a targeting protein in the ubiquitination–proteasome degradation pathway in Arabidopsis, and HISTONE DEACETYLASE 2C (HD2C) is one of its interacting partners. It has been shown that HOS15 interacts with histone deacetylase 2C (HD2C), and together, they are associated with the promoters of cold-responsive COR genes [33]. It was suggested that cold induces HOS15-mediated chromatin modifications by degrading HD2C. Loss-of-function *hos15* mutant plants were sensitive to cold, regardless of cold acclimation, as opposed to *hd2c* mutants, which showed freezing tolerance at the level of wild-type plants. This implied that the histone H3 deacetylating activity of HD2C negatively regulates cold acclimation gene regulation, and HOS15 counteracts this negative regulation [27]. Histone acetylation also plays an important role in cold stress response in plants, and it has been shown that histone acetylation is enriched in the gene body of cold-responsive genes [33], which is regulated by histone acetyltransferases (HATs) and histone deacetylases (HDACs). Histone acetylation was also induced upon cold treatment in the promoter regions of some COR genes [34]. In another study, cold stress treatment in maize was shown to lead to the upregulation of HDACs, which resulted in the deacetylation of H3 and H4 and the activation of heterochromatic tandem repeats. This caused a reduction in DNA methylation and histone dimethylation (H3K9me2) at the targeted region of the genome [35,36]. Cold stress treatment on maize seedlings reported genome-wide DNA methylation, except only in a 1.8 kb segment (ZmMI1). The ZmMI1 segment is representative of a stress response gene and is normally methylated, but under cold stress conditions, it experiences demethylation. The hypomethylation from cold stress was maintained seven days after the cold treatment was lifted [36]. Studies in crops have also shown the role of epigenetic changes in low-temperature-induced dormancy. Chilling temperature reduced total methylation, which then resulted in restarting growth and subsequent fruit setting [37,38].

## 4. Epigenetic and Salt Stress

Salinity stress has serious negative impacts on plant life, mainly because of sodium ion toxicity, osmotic stress, and secondary stresses such as oxidative damages [27]. DNA methylation plays an important role in regulating the expression of salt-responsive genes. Studying variations in global DNA methylation levels in salt-tolerant and salt-sensitive rice varieties in response to salt stress revealed that under salinity, stress promoter and gene body methylation play important roles in regulating gene expression in a genotype- and organ-specific manner. Hypomethylation was reported in response to high salinity, which correlated with the differential expression of the DNA demethylase (DRM2) gene, which was upregulated under salt stress in salt-sensitive but not salt-tolerant cultivar [39]. The results suggested the potential impact of differential DNA methylation patterns on salt stress tolerance in plants.

HIGH-AFFINITY K+ CHANNEL1 (HKT1), which mediates Na+ influx in plants, is a transporter that coordinates with the SALT OVERLY SENSITIVE (SOS) pathway, resulting in salt resistance [40]. Mutation of HKT1 could suppress the salt-hypersensitive phenotype of SOS plants (Rus et al. 2001). In wild-type Arabidopsis, a putative small RNA target region was identified at about 2.6 kb upstream of the ATG start codon HKT1, and its promoter region was shown to be heavily methylated in all sequence contexts [41]. In the small RNA biogenesis mutant *rdr2*, CHG and CHH methylation of this region is significantly reduced, whereas CG methylation is unchanged, implying that non-CG methylation in this region is mediated by small RNAs. In the *met1* mutant, which is DNA methylation deficient, cytosine methylation in all sequence contexts is significantly reduced in the putative small RNA target region and is hypersensitive to salt stress, while the *rdr2* mutant that lost non-CG methylation has normal salt sensitivity. Thus, hypermethylation of the promoter region of the HKT1 gene in all contexts can prevent transcription, whereas non-CG methylation could regulate the expression of HKT1 and help with the adaptation of plants to salt stress [27]. The HKT1 gene body is shown to have a high level of H3K27me3, and salinity upregulates HKT1, which is suggested to be due to the removal of H3K27me3 [42]. In the Arabidopsis RdDM mutant, DNA methylation in this region was decreased, which caused the upregulation of HKT1, implying that RdDM negatively regulates AtHKT1 gene expression. Studies on wheat also showed similar results, where high-level methylation resulted from salt stress that correlated with the downregulation of HKT genes [43]. Different wheat genotypes responded differently to salt stress, which could be explained by the different expression levels of high-affinity potassium transporters (HKTs) that regulate through genetic and/or epigenetic mechanisms [44]. The gene body of HKT2 genes in the wheat genotypes is reported to have variation in the level of 5-methyl cytosine content in different genotypes, tissues, and growth/stress conditions, which was significantly increased by salt stress and correlated with the downregulated expression of HKT2 genes. In another study, Wang et al. [45] reported distinguished DNA methylation patterns in salt-tolerant wheat cultivar and its progenitor after salt stress treatment, which was suggested to be linked to the differential expression levels of high-affinity potassium transporters (HKTs) regulated by genetic and/or epigenetic mechanisms.

MYB74, a member of the R2R3-MYB gene family, is a salt-induced transcription factor and, similar to HKT1, the promoter of MYB74, is normally highly methylated by the RdDM pathway, hence MYB74 shows a low expression level in normal conditions. On the other hand, under high salinity conditions, methylation and 24-nt siRNA levels become very low at MYB74, which is associated with the higher expression of MYB74 as well [4]. DNA methylation in CG and CHH contexts and siRNA target sites were found in the MYB74 promoter region. Five 24-nt siRNAs were predicted to target a small region of the MYB74 promoter, and their accumulation showed a strong reduction under salt stress. Therefore, a reduction in DNA methylation and expression of MYB74 transcripts under salt stress was suggested to result from the decrease in these 24-nt siRNAs [4].

Histone modification dynamics, which cause variations in chromatin structure, also play important roles in plant responses to salinity stress. The coordination between histone acetyltransferases (HATs) and histone deacetylases (HDACs) is important for the adaptation of plants to changing environments [46]. Salt stress usually causes the deposition of active histone marks such as H3K9K14Ac and H3K4me3, and it decreases the deposition of repressive histone marks such as H3K9me2 and H3K27me3 on salt-tolerance genes [47]. HD2 proteins are plant-specific histone deacetylases. Four HD2 proteins, HD2A, HD2B, HD2C, and HD2D, have been identified in Arabidopsis. It has been reported that the expression of HD2A, HD2B, HD2C, and HD2D is repressed by abscisic acid (ABA) and also by high salinity. Luo et al. [48] reported that compared with wild-type plants, *hd2c* mutants were more sensitive to ABA and NaCl during germination and showed decreased tolerance to salt stress. This supports HD2C’s role in the ABA and salt stress response. Moreover, it was demonstrated that HD2C physically interacted with HISTONE DEACETYLASE 6 (HDA6) and bound to histone H3 to repress the ABA-responsive genes ABA1 and ABI2 in normal conditions [48]. They concluded that HD2C associates with HDA6 and regulates gene expression through histone modifications. In another study using Arabidopsis mutants, it was shown that the transcriptional adaptor ADA2b (a modulator of histone acetyltransferases activity) plays an important role in salt stress susceptibility. ADA2b upregulated salt-responsive genes through the locus-specific acetylation of histones H4 and H3 [49]. Another histone modification that can be induced by salinity treatment is H3 Ser-10 phosphorylation, which is usually related to chromatin density. Studying the nucleosomal response of plant cells to high salinity in tobacco and Arabidopsis cell lines showed the rapid upregulation of histone H3 Ser-10 phosphorylation, followed by the upregulation of H3 phosphoacetylation and histone H4 acetylation. The observed three types of nucleosomal responses were significantly associated with the expression of stress-type-specific genes [50]. Histone deacetylase 6 (HDA6) is important for H3K4me3 of salt-responsive genes, and similar to ada2b-1, mutations in this gene result in increased salt susceptibility and reduced abiotic stress-tolerance gene expression in Arabidopsis [51]. It should be noted that histone modifications are reversible, and crosstalk between histone acetylation and DNA methylation makes the plant responses to stress more complicated. For example, studies of the high salinity effect in soybean suggested a combined role for DNA methylation and histone modifications in the activation/repression of some stress-inducible transcription factors [52]. Taken together, salt stress has an important impact on genome-wide DNA methylation and histone modifications, and such modifications are linked to each other to help plants against salt stress [53].

## 5. Epigenetic and Drought Stress

The phytohormone ABA in plants is synthesized in response to drought stress, which then enables ABA to help with drought resistance [54]. ABA controls multiple developmental phases such as germination, stomatal closure, and root growth and plays a key role in growth arrest in seedlings when ABA-dependent transcription factors alter transcriptional expression patterns in a reversible manner. The regulatory pathway of ABA signaling has been introduced before [28], and the epigenetic mechanisms behind ABA stress responses have been widely studied (e.g., [55,56]). It has also been shown that the expression of genes related to drought stress is in close relationship with the change in histone dynamics (e.g., [26]). Histone modifications play a key role in ABA responses during seedling development [57]. BR-activated BES1 forms a transcriptional repressor complex with TPL-HDA19 (BRI1-EMS-SUPPRESSOR 1 (BES1)-TOPLESS (TPL)-HDA19 histone deacetylation complex), which then directly facilitates the histone deacetylation of ABI3 chromatin, leading to the transcriptional repression of ABI3 and consequently ABI5, major ABA signaling regulators in early seedling development. It is suggested that the BR-activated BES1-TPL-HDA19 repressor complex affects the epigenetic silencing of ABI3, suppressing the ABA signaling output during early seedling development [57]. In addition to histone modification, ROS1-dependent DNA demethylation has also been shown to regulate the expression of a subset of ABA-inducible genes [58].

Transcriptional expressions of drought stress genes are aligned with histone modifications and nucleosome density [26]. Severe drought stress causes an increase in H3K4me3 and H3K9Ac and a reduction in nucleosome density on stress-responsive genes compared with less severe dehydration. Therefore, epigenetic dynamics seem to depend on the amount of stress. Recovering from drought stress shows a rapid decrease in H3K9Ac and the removal of RNA polymerase II from the drought-stress-upregulated genes, while H3K4me3 decreases gradually [27]. Drought stress increases the deposition of H3K4me3 within the gene body region of NINE CISEPOXYCAROTENOID DIOXYGENASE 3 (NCED3), which is a key enzyme involved in ABA synthesis, and it also causes the enrichment of the NCED3 gene [59]. Another study reported that under drought stress, H3K4me3 and H3K9Ac levels in the promoter regions of drought-responsive genes such as RD29A, RD29B, RD22, and RELATED TO AP2.4 (RAP2.4) increase and result in the upregulation of these genes [60]. Interestingly, the abundance of histone marks within these genes depends on the severity of drought stress. H3K4me3 and H3K9Ac levels increase a lot more under severe drought stress compared to less intense drought stress [61]. When the drought stress is over and after plants recover from dehydration, H3K4me3 and H3K9Ac are removed from the drought genes, which, in the case of H3K9Ac, happens quicker than H3K4me3 [61]. Ramirez-Prado et al. [62] reported that the reduction in H3K27me3 deposition within the gene body of drought-response transcription factors is important for drought tolerance in *Arabidopsis*.

An earlier study using Arabidopsis investigated the role of the SNF2/Brahma-type chromatin remodeling protein CHROMATIN REMODELING 12 (CHR12) in plants dealing with severe environmental stresses and showed that this chromatin-remodeling gene plays an important role in mediating the temporary growth arrest of Arabidopsis that is induced upon the perception of stresses, including drought and heat stresses [63]. Histone demethylases are involved in the fine tuning of SnRK2.8 kinase via the ABI3 transcription factor. Wu et al. [64] showed that ABA-mediated growth arrest in *A. thaliana* is controlled by the histone demethylases JUMONJI-C domain-containing protein 30 (JMJ30) and JMJ32. During the postgermination stage (2–3 days after germination), the ABA-dependent transcription factor ABA-insensitive3 (ABI3) activates the expression of JMJ30 in response to ABA. JMJ30 can remove the repressive histone marker H3K27me3 from the promoter region of SnRK2.8 and activate SnRK2.8 expression. SnRK2.8 kinase, in turn, activates the expression of ABI3 and is responsible for JMJ30- and JMJ32-mediated growth arrest [64]. These findings highlight the key role of histone demethylases in plant drought adaptation. Another study of maize showed that a miniature inverted-repeat transposable element (MITE) inserts in the promoter of a NAC gene and represses its expression through RdDM and H3K9me2 deposition [65]. Drought stress memory via the histone modification of drought stress genes has also been reported [46]. Studies on *A. thaliana* have shown that increasing levels of H3K4me3 and H3K9Ac on the promoter and H3K23 and H3K27 acetylation on the gene body region induces the expression of drought-stress genes [66].

Khan et al. [67] showed that Arabidopsis STRESS RESPONSE SUPPRESSOR1 (STRS1) and STRS2-overexpressing lines displayed lower expression of stress-induced genes. They reported that, similar to ABA treatment, the malfunctions of several RdDM proteins and HD2C resulted in the mislocalization of STRS2 and STRS1, respectively. Furthermore, heterochromatic RdDM target loci displayed reduced DNA methylation and increased expression in the *strs* mutants, which suggested that the STRS proteins are involved in the epigenetic silencing of gene expression to suppress stress response in Arabidopsis. The Arabidopsis trithorax-like factor ATX1, which trimethylates histone H3 at lysine 4 (H3K4me3), stimulates the transcription of multiple genes involved in dehydration stress [59]. Plants with an atx1 mutation showed decreased germination rates and more rapid transpiration by their leaves due to higher stomatal apertures, which decreased their tolerance to dehydration stress. This deficiency was caused partly by reduced ABA levels in atx1 plants (40% compared to the wild-type plants) resulting from decreased transcript levels from NCED3, which encodes a key enzyme controlling ABA biosynthesis. Dehydration stress increased ATX1 binding to a promoter region of NCED3, and ATX1 was required for the increased levels of NCED3 transcripts and nucleosomal H3K4me3 that occurred during dehydration stress. Therefore, the levels of H3K4me3 at the NCED3 promoter region were increased by dehydration stress. The H3K4me3 levels at the representative drought-stress-responsive genes showed a correlation with their expression levels, and genes downregulated in atx1 plants showed reduced levels of H3K4me3 [59].

In addition to histone modifications, DNA methylation also contributes to drought resistance in plants. Many studies are available on the changes of DNA methylation levels caused by drought stress in annual, herbaceous plants [10,68,69], but few reports are available for perennial woody plants. Studying *Populus trichocarpa*, Liang et al. [70] reported that drought stress changes DNA methylation levels, and therefore, it changes the expression patterns of many drought-stress-response genes. Genome-wide methylation levels of methylated cytosines were significantly higher under drought stress compared with control plants, which were also positively associated with gene expression, and intense methylation resulted in gene silencing. Reduced DNA methylation and transcriptome expression after drought treatment were found in genes encoding for some TFs, and an increase in DNA methylation and gene expression was found in genes coding for other TFs. Therefore, changes in DNA methylation could regulate the expression of drought-response genes at the genome-wide scale. In another study, drought stress showed extensive remodeling of DNA methylation patterns in poplar, and DNA methylation at repetitive elements is shown to play an important role in controlling the expression of neighboring genes [70]. This is also reported in maize, where TEs were enhancers of stress-responsive genes [71]. DNA methylation patterns and their association with abiotic stress were shown in the tree crop *Hevea brasiliensis* as well [72]. For *Quercus ilex* trees, it was reported that the percentage of hypermethylated loci increased and that of fully methylated loci decreased when they were exposed to drought [73]. Xu et al. [74] reported the demethylation of TEs under drought stress in apple (*Malus domesticus*). In another study in poplar [75], hypomethylation in gene bodies was observed under drought stress, followed by hypermethylation after withdrawal of drought stress. The opposite effect was observed for TEs [75]. Hypomethylation and therefore downregulation of some hormone-responsive genes were observed after the drought stress was lifted, which suggested that when plants experience drought stress, crosstalk between DNA methylation and enzymes that inhibits the expression of certain genes through histone modification (known as polycomb complexes) happens [76]. The *Malus domestica* apple tree is less tolerant to drought compared with its wild relative (*M. prunifolia*). It is suggested that the higher drought tolerance of *M. prunifolia* comes from the lower promoter methylation and higher expression of the gene DREB2A, which is a transcription family member linked to plant resistance and heat and drought stress [77]. Therefore, methylation of this promoter region may play a role in drought resistance in *M. prunifolia*. Wang et al. [10] detected genotypic specific patterns of drought-induced DNA methylation sites in rice, where at 70% of the sites, epigenetic changes were reversed to prestress status after recovery, and at 29% of sites, drought-induced DNA demethylation/methylation changes remained even after recovery. They also found a significant level of developmental and tissue specificity of DNA methylation variation and suggested drought-induced DNA methylation variations in the rice genome can be considered a very important regulatory mechanism for adaptation in rice plants. Another study using rice drought-tolerance/susceptible cultivars also reported the role of DNA methylation in drought resistance. The susceptible varieties showed hypomethylation under drought conditions, while the tolerant varieties showed hypermethylation. The dynamics of the methylation pattern also affected the expression of drought-response genes [78]. In summary, chromatin changes caused by both histone modifications and DNA methylation play important roles in drought tolerance in plants [7].

## 6. Epigenetic, Nutrient, and UV Stress

Plants have evolved to adapt to fluctuating amounts of nutrients [79]. Nitrate (N) uptake is under systemic feedback repression by the N satiety of plants. High N supply represses the expression of a root nitrogen transporter, NRT2.1, through a negative feedback loop mediated by High-nitrogen-insensitive 9 (HNI9), a critical factor in the deposition of repressive H3K27me3 marks at the NRT2.1 gene [80]. High nitrogen-insensitive 9-1 (*hni9-1*) mutants are impaired in that high N supply feedback repression. The gene repression requires HIGH-NITROGEN-INSENSITIVE 9 (HNI9) that encodes INTERACT WITH SPT6 (IWS1), a component of the RNA polymerase II complex and repression of NRT2.1 transcription by high N supply is associated with an HNI9/AtIWS1-dependent increase in histone H3K27m3 at the NRT2.1 locus [80]. Therefore, post-translational chromatin changes affect nutrient uptake in plants.

Histone methylation and histone acetylation have also been proposed to affect iron homeostasis in plants [81,82]. Fan et al. [81] reported that H4R3 symmetric dimethylation (H4R3sme2) negatively regulates iron homeostasis, and Xing et al. [82] showed general control nonrepressed protein 5 (GCN5), a histone acetyltransferase, binds to the promoters of FERRIC REDUCTASE DEFECTIVE 3 (FRD3), an iron-related gene, and modulates the acetylation levels of H3K6 and H3K14, which then facilitate iron translocation. In addition, H3K4me3, histone acetylation, and histone variant H2A.Z have all shown to play important roles in phosphate (Pi) deficiency [83,84,85]. The Arabidopsis *gcn5* mutant impairs the iron translocation from the root to the shoot [82]. In addition to histone modifications, Pi deficiency has shown massive remodeling of global DNA methylation as well [86] and also affected the transcription level of DNA methylases genes such as MET1, DRM1, and DRM2 [87]. Some main sulfate-responsive genes also are reported to be dependent on DNA methylation regulation [88]. In addition, differential symmetric DNA methylation has also been shown to be associated with the upregulation of some Zn-deficiency-response genes [89].

Lang-Mladek et al. [90] reported immediate and heritable changes in the epigenetic control of a silent reporter gene in Arabidopsis in response to UV-B stress, which was associated with changes in chromatin conformation and histone H3 acetylation but did not involve adjustments in DNA methylation. In another study, Pandey and Pandey-Rai [91] reported DNA hypomethylation in response to UV radiation in *Artemisia annua*, which produces artemisinin, a sesquiterpene required for malaria treatment. They reported expression of DOUBLE-BOND REDUCTASE 2 (DBR2), a key regulatory gene of artemisinin biosynthesis- in response to UV-B treatment, through inducing DNA demethylation in the DBR2 promoter region, which contains WRKY transcription factor binding sites [91].

## 7. Epigenetic and Stress Memory

Because of their sessile nature, plants spend their entire lives in one fixed spot and have to quickly adapt to any changes in their environment. Adaptation to global warming requires plants to use strategies such as dynamic changes in gene expression through epigenetic mechanisms such as loss or gain of DNA methylation, which might even transmit to the next generation, which then becomes a source of inherited phenotypic variation in response to stress without changing the DNA sequence. This is called stress memory, which enables plants to react more effectively in the face of recurring stress and even strengthen the next generation for possible future assault [92]. After plants are exposed to stress for a period of time, they are able to retain the stress response information to some degree and for at least some specific stress-responsive genes. This ability will ensure their adaptation to similar future stress more quickly and efficiently. The ability of plants to encounter recurring stress was named stress priming [35]. In fact, priming has been defined as a way for plants to take advantage of current abiotic stress cues in order to develop a quicker, stronger, and more efficient coping response to future recurring stress [93].

It is known that stress memory is closely correlated with epigenetic changes [5]. Many studies have shown that stress treatment can induce changes in the chromatin status of stress-responsive genes, which can be preserved until after recovery or even in the offspring [31,42,94]. Genome-wide epigenetic changes are reported to be associated with gene expression differentiation in response to stress, and they both may return to the prestress state after the stress is removed. However, in addition to priming memory during a plant’s life span, studies have suggested that DNA sequence-independent epigenetic modification could transmit to the next generation, which is called “transgenerational memory” [95]. This means that some epigenetic modifications are maintained and transferred to the next generation as stress transgenerational memory, which ensures plasticity and adaptation in plants and provides them with a better balance between survival and reproduction [96]. To study the role of epigenetic mechanisms in long-term adaptation, Zheng et al. (2017) established two rice epimutation accumulation lines by applying drought conditions to 11 successive generations of two rice varieties. They reported that drought adaptability was improved because of the multigenerational drought experience. They found that many drought-induced epimutations (>40%) preserved their changed DNA methylation status in younger generations. In addition, genes related to transgenerational epimutations directly participated in stress-responsive pathways, and their DNA methylation patterns were affected by multigenerational drought. The progenies also showed a decrease in water consumption and maintained the yield. Therefore, it was suggested that drought-response DNA methylation memory has an impact on plant adaptation to drought stress conditions [97].

Another study reported that DNA methylation is involved in the transgenerational memory of the response to heavy metals stress in rice [95]. They showed that heavy metal-transporting P-type ATPase genes (HMAs) were upregulated in response to heavy metal stress, and the transgenerational memory of gene expression was observed after returning to normal conditions. They also reported changes in the DNA methylation of a Tos17 retrotransposon in response to heavy metal stress, which showed transgenerational inheritance for three generations [95]. It is known that heat stress could activate the transcription of ONSEN retrotransposon, and heat-induced ONSEN accumulation was stimulated in the mutants of small interfering RNAs [98]. Even though, after stress, both ONSEN transcripts and extrachromosomal DNA were no longer detected after 20–30 days, a high frequency of new ONSEN insertions was observed in the progeny of stressed plants deficient in siRNAs. Therefore, they suggested that stress memory could be maintained in plants with compromised siRNA biogenesis. In apomictic dandelions (*Taraxacum officinale*), the global DNA methylation pattern in progenies was changed when the parental plants were imposed with environmental stress and provided evidence for the existence of transgenerational memory effects [99].

In addition to DNA methylation dynamics, histone modification is also directly relevant to intergenerational and/or intragenerational stress memory in plants [27]. H3K4me3 has been shown to be an epigenetic mark that is associated with transcriptional stress memory [35]. Liu et al. [100] also showed that H3K4me3, and not H3K27me3, could be an epigenetic memory mark for drought-stress-responsive genes. In another study, Sani et al. [42] investigated long-term memory for salinity stress in Arabidopsis plants and reported that after a recovery phase, plants that were primed by mild salt stress showed less salt uptake and higher drought tolerance compared with control plants. They showed that salt treatment priming led to a decrease in H3K27me3 at the edges of H3K27me3-enriched islands in the whole genome, resulting in the shortening and fractionation of H3K27me3 islands that faded over time but still existed after a ten-day growth period in control conditions. Several genes with priming-induced differences in H3K27me3 showed changes in transcriptional responsiveness to the second stress treatment. They then analyzed genome regions that varied in the abundance of histone methylation between primed and nonprimed plants and reported higher methylation levels in the primed plants for H3K4me2 and H3K4me3. However, most of the differential H3K27me3 regions showed lower methylation levels in the primed plants. They concluded that transient hyperosmotic stress by young plants is stored in a long-term somatic memory in the shape of different chromatin statuses and therefore different expressions of stress-responsive genes.

Another study revealed that a heat-stress-responsive gene HSP22.0 is involved in heat stress memory, and its expression increased after heat stress [17]. Heat stress memory has been shown to be associated with the accumulation of H3K4 methylation at stress-memory-related loci. The accumulation of heat marks them as recently transcriptionally active, and the high accumulation of H3K4 methylation is associated with hyperexpression of the gene upon recurring heat stress. This transcriptional memory and the sustained accumulation of H3K4 methylation depend on a heat stress memory transcription factor called “HSFA2”. Interestingly, HSFA2 is associated with memory-related loci transiently during the early stages following heat stress. In summary, Lamke et al. [17] concluded that heat stress could cause the deposition of active histone marks in the HSP22.0 genes and reported that transcriptional memory after heat stress is associated with sustained H3K4 hypermethylation and depends on the HSFA2- transcription factor. In another study, when Arabidopsis plants were infected with bacteria, their progeny showed more tolerance to secondary infection of oomycete than that of the progeny of control plants [101]. Inherited priming was shown to be caused by epigenetic mechanisms. The upregulation of defense genes was correlated with histone acetylation in the promoter region, whereas downregulation of the genes was linked to a higher level of the repressive epimark H3K27me3. Interestingly, plants that were defective in CHG- DNA methylation mimicked the effects of transgenerational priming [102]. Thus, it was suggested that transgenerational stress memory might be modulated by CHG- DNA demethylation, which might involve a complex mechanism and a series of epigenetic changes wherein the biotic stress triggers loss of repressive epimark that then activates epimark. All these studies show that priming treatment may change the epigenomic pattern and therefore create epigenetic stress memory. Stress memory can also be considered a tool to improve stress tolerance in crop plants either via priming or by targeted modifying of the epigenome.

## 8. RNA Methylation and Abiotic Stress Responses in Plants

In addition to DNA methylation, post-transcriptional RNA modifications are also known as plant epigenetic regulators [103,104]. N6-methyladenosine (m6A) and 5-methylcytidine (m5C) are two types of RNA methylations. They are the most common mRNA modifications in eukaryotes, with m6A being more prevalent in both plants and animals [105]. The level of m6A varies according to the activity of the cellular factors and enzymatic machinery, named “writers (methyltransferase)”, which catalyze the methylation process; “erasers (demethylase)”, mediating adenosine demethylation; and “readers (RNA-binding protein)”, introducing, deleting, and interpreting specific methylation marks on mRNAs, respectively [106]. Our knowledge of writers, readers, and erasers in plants is far behind that of their animal counterparts, and the identity and functions of these factors in plants are currently not very clear. Although recent studies have reported about the roles of m6A writers in plant growth and development [107,108], deep studies about their impact on plant response to abiotic stresses are lacking. Hu et al. [106] systematically identified potential m6A writers, readers, and erasers in *A. thaliana* and rice (*Oryza sativa*) by searching their homologous sequences against animal databases. They analyzed publicly available microarray data and reported that expressions levels of writers in Arabidopsis and rice are differently affected by diverse abiotic stresses. In Arabidopsis, levels of most m6A writer components were not significantly modulated by abiotic stresses or in some cases were only marginally increased by only cold and heat stress. In rice, the level of some writers was increased by cold stress, whereas the levels of others were decreased by cold, drought, or salt stress. The expression of m6A writer components under both normal and stress conditions led to the conclusion that m6A methylation in plants may affect both the development and stress responses. In another study, Anderson et al. [109] reported differential mRNA m6A methylation in Arabidopsis upon salt treatment and identified a strong association between m6A methylation and salinity stress response. A study using Arabidopsis mutants of *m6A* writer components showed the important role of m6A methylation in salt tolerance [110]. It was reported that one of the m6A writer components named VIRILIZER (VIR) modulates the expression of many salt-stress-response genes. It was also reported that VIR-mediated m6A mRNA methylation is associated with the mRNA stability of salt-stress-negative regulators. The findings suggested a link between m6A methylation and mRNA stability during adaptation to stress [110].

Erasers are the least studied among RNA methylation factors in plants. However, new information is being collected [111]. The α-ketoglutarate-dependent dioxygenase homolog (ALKBH) protein family is one of the known erasers in plants. Thirteen members of them have been identified by bioinformatic analysis in Arabidopsis [112], and only a few of them have been studied. It has been shown that ALKBH9A is highly expressed in roots under salt stress but not in response to ABA, and its level of expression is much lower than ALKBH9 and ALKBH10 under normal conditions [113]. ALKBH10A is downregulated under heat stress [114], whereas ALKBH10B is upregulated in response to karrikins [115]. These studies imply a potential role for ALKBHs in stress responses. In their analyses, Hu et al. [106] reported that expression levels of ALKBH members were marginally up- or downregulated under different abiotic stress factors. For example, ALKBH1 was upregulated under drought, cold, or ABA treatment in rice, whereas ALKBH6, ALKBH8B, and ALKBH10A were all downregulated by drought, ABA, or cold. They concluded that ALKBHs could potentially be important for abiotic stress responses, although this requires more investigations.

Adenosine methylation of mRNA results in its remodeling and therefore increases the chances for its binding with specific reader proteins, which are usually members of the YTH family [116]. Although several RNA methylation reader proteins (interpreting m6A marks) have been reported in animals, only three m6A reader proteins (from YTHD family) are identified in Arabidopsis [107,117,118]. Different expression levels of proteins belonging to the YTH domain family in response to stress factors are reported that suggest their role in plants’ reaction to stressful conditions (e.g., YTHD09). Cytoplasmic-localized YTHD09 relocates to stress granules upon heat stress [118,119,120]. This is also supported by studies that introduced some YTH domain proteins from apple into Arabidopsis plants, and as a result, higher tolerance to salinity and drought in Arabidopsis was found [121]. It has also been shown that the expression of different members of the YTHD family in Arabidopsis and rice is either increased or decreased under different abiotic stresses [106,122]. The fact that m6A reader proteins are more responsive to abiotic stresses than writers and erasers, or at least this is what appears to be based on our knowledge at the time of writing this review, implies that under stress conditions, the decoding and interpreting of methylation marks are much more important than methylation and demethylation, which might help with the adaptation of plants to stresses. Future studies are needed to identify the function of reader proteins in RNA metabolism and its impact on stress tolerance in plants.

Even though m6A is the most common mRNA methylation, other methylated ribonucleotides might also have significant effects on the functioning of plant cells [123]. In fact, m5C in mRNA has been reported in *A. thaliana*, *Zea mays*, *Oryza sativa*, *foxtail millet*, and *Medicago truncatula*. This modification occurs with the activity of tRNA-specific methyltransferase 4 (TRM4) and various external factors, such as drought, heat, and treatment with phytohormones. A reduced level of m5C is correlated with reduced root length, inhibited cell proliferation, and higher sensitivity to oxidative stress, which suggests the role of m5C in the regulation of both plant development and oxidative responses in plants [124,125]. Although cytosine methylation (m5C) in DNA has been studied for many years, its functions in RNAs are just starting to be noticed. Overall, it is a less common modification of mRNA than m6A methylation. In their analyses, Hu et al. [106] found two enzymes responsible for m5C RNA methylation in Arabidopsi. Even though their expression patterns suggested the potential roles of m5C writers in abiotic stress response, the relevance of m5C methylation to abiotic stress responses was not clear and needed more investigation.

## 9. Conclusions

In summary, epigenetic marks on stress-induced genes dynamically change and therefore affect the accessibility of chromatin and the expression of those genes at the transcriptional or translational level. Epigenetic changes such as DNA methylation, histone modifications, chromatin remodeling, histone variants, and long noncoding RNAs (lncRNAs) may all be involved in the various regulatory mechanisms of abiotic stress responses. The important role of epigenetic modifications in regulating gene expression, and also their ability to transfer to the next generation, makes them a unique adaptation tool for plants. The phenotypic plasticity caused by epigenetic variation, which, in turn, is through changes in gene expression, will affect fitness and eventually natural selection in plants. Unlike classic DNA sequence mutations, epimutations can happen at much shorter times, and even though they are stable, they are also mostly reversible, which makes them a perfect tool for a quick emergency response to unpredictable environmental stresses. It should also be noted that epigenetic variations usually depend on the underlying genetic variation, and these two aspects need to be studied in parallel. Future studies are needed for a deeper understanding of the epigenetic mechanisms behind chromatin alterations and the subsequent transcriptional regulations that affect plants’ response to environmental stresses. The mechanism of inherited stress memory also needs more attention.
